# Rituximab-Based Treatment, HCV Replication, and Hepatic Flares

**DOI:** 10.1155/2012/945950

**Published:** 2012-08-05

**Authors:** Evangelista Sagnelli, Mariantonietta Pisaturo, Caterina Sagnelli, Nicola Coppola

**Affiliations:** ^1^Section of Infectious Diseases, Department of Public Medicine, Second University of Naples, 80131 Naples, Italy; ^2^Division of Infectious Diseases, AORN Sant'Anna e San Sebastiano di Caserta, 81100 Caserta, Italy; ^3^Department of Clinical and Experimental Medicine and Surgery “F. Magrassi e A. Lanzara”, Second University of Naples, 80131 Naples, Italy

## Abstract

Rituximab, a chimeric mouse-human monoclonal antibody directed to the CD20 antigen expressed on pre-B lymphocytes and mature lymphocytes, causes a profound B-cell depletion. Due to its peculiar characteristics, this drug has been used to treat oncohaematological diseases, B cell-related autoimmune diseases, rheumatoid arthritis, and, more recently, HCV-associated mixed cryoglobulinaemic vasculitis. Rituximab-based treatment, however, may induce an increased replication of several viruses such as hepatitis B virus, cytomegalovirus, varicella-zoster virus, echovirus, and parvovirus B19. Recent data suggest that rituximab-based chemotherapy induces an increase in HCV expression in hepatic cells, which may become a target for a cell-mediated immune reaction after the withdrawal of treatment and the restoration of the immune control. Only a *few* small studies have investigated the occurrence of HCV reactivation and an associated hepatic flare in patients with oncohaematological diseases receiving R-CHOP (rituximab, cyclophosphamide, doxorubicin, vincristine, and prednisone). These studies suggest that the hepatic flares are frequently asymptomatic, but life-threatening liver failure occurs in nearly 10% of cases.

## 1. Introduction

Hepatitis C virus (HCV) is responsible worldwide for chronic hepatitis, liver cirrhosis, and hepatocellular carcinoma [1]. Besides its hepatotropic characteristics, HCV is also a lymphotropic virus [2, 3] responsible for HCV-related B-cell non-Hodgkin lymphoma (NHL) [4–6], immune-mediated extrahepatic manifestations, mixed cryoglobulinaemic vasculitis [7–12], and the presence in serum of rheumatoid factor and autoantibodies [13–15]. 

In the last decade, rituximab, a chimeric mouse-human monoclonal antibody directed to the CD20 antigen expressed on pre-B lymphocytes and mature lymphocytes [16], has been used increasingly for treating patients with haematological diseases including CD20-positive B-cell NHL [17]. Rituximab causes a profound B-cell depletion, peripheral blood B lymphocytes becoming undetectable after a single infusion, with a complete B-cell recovery from 6 to 9 months after the discontinuation of treatment [18, 19]. Due to its peculiar characteristics, this drug has also been used for treating B cell-related autoimmune diseases [20], rheumatoid arthritis, and, more recently, HCV-associated mixed cryoglobulinaemic vasculitis [21, 22]. 

It is well known that rituximab-based chemotherapy is frequently followed by a reactivation of viral infections and correlated diseases. A frequent increase in viral replication under R-CHOP (rituximab, cyclophosphamide, doxorubicin, vincristine, and prednisone) was demonstrated by Aksoy et al. for several viruses including hepatitis B virus (HBV), cytomegalovirus, varicella-zoster virus, echovirus, and parvovirus B19 [23]. Worthy of mention is HBV infection, both overt and occult, which in the absence of a specific prophylaxis or treatment frequently reactivates during or after R-CHOP, with a mortality rate close to 20% and death being due to liver failure or to an unfavourable progression of the underlying haematological disease once R-CHOP has been discontinued because of a hepatic flare [24–33].

### 1.1. Studies on HCV Reactivation and Hepatic Flares due to Rituximab-Based Chemotherapy 

According to the fragmentar data available, R-CHOP can induce an increase in HCV replication in oncohaematological patients [34, 35], a biological event associated to the development of hepatic flares in some studies [36–41]. A reasonable explanation for this association may be that R-CHOP induces an increase in HCV expression in hepatic cells, which may become a target for a cell-mediated immune reaction after the discontinuation of treatment and the restoration of the immune control. This hypothesis, however, requires support from further studies with a longer follow up to clarify the relationship between HCV reactivation and the occurrence of a hepatic flare and to assess the clinical impact of flares.

The uncertainty dominating this topic arises from the inconsistent data available and from the marked differences in the studies as regards the design, age of the patients, type and stage of the oncohaematological diseases, type of chemotherapy used, and stage of liver disease. In addition, the criteria used to define HCV reactivation and a hepatic flare differ from study to study.

Some of these papers are case reports. Akosy et al. described HCV reactivation not associated to a hepatic flare in a patient with HCV-related cirrhosis and NHL treated only with rituximab [34]. Nooka et al. published a similar observation in a patient with diffuse large B-cell lymphoma (DLBCL) and HCV infection who experienced an asymptomatic HCV reactivation during R-CHOP [35]. Hsieh et al. described an HCV reactivation with a hepatic flare in a patient with DLBCL receiving R-CHOP [41], and Lake-Bakaar reported a case of HCV reactivation in a patient with HCV-related mixed cryoglobulinaemia who developed a hepatic flare 2 weeks after starting rituximab treatment [42]. 

Only a few studies evaluated both HCV reactivation and the development of a hepatic flare in small series of patients with oncohaematological diseases receiving R-CHOP (Table 1). In a prospective study on 8 anti-HCV/HCV RNA-positive patients undergoing chemotherapy, HCV replication was determined both in plasma and peripheral blood mononuclear cells (PBMC). In this study, Coppola et al. [40] found an increase in HCV RNA of at least 1.5 log IU/mL in plasma and of at least 1.1 log IU/mL in PBMC of the 7 patients receiving rituximab and corticosteroid-based chemotherapy, whereas no change was observed in the one patient treated with rituximab-sparing chemotherapy. In this study, the patients with HCV reactivation showed a hepatic flare 3–5 months after treatment was discontinued; this was life threatening only in one patient who had compensated cirrhosis at the baseline and developed a grade-3 hepatic flare, ascites, and portosystemic encephalopathy with a progression of Child-Pugh score from A6 to B9 (Table 1).

In a retrospective study on 131 patients with HCV infection and NHL treated with rituximab and prednisone-based chemotherapy, Ennishi et al. [36] described a hepatic flare in 36 patients (27%), of these 131, 34 with DLBCL showed HCV reactivation during the follow up, but the prevalence of hepatic flares in these 34 patients was not reported (Table 1). In nearly 10% of cases, the hepatic flares were life threatening and some patients died of liver failure. The retrospective nature of the study, however, suggests caution in accepting these data as conclusive. 

In a retrospective study, Marignani et al. [39] described 3 patients with HCV infection and NHL treated with R-CHOP therapy. Two of these patients experienced HCV reactivation and showed a hepatic flare, which was symptomatic in one of the two, after chemotherapy was discontinued (Table 1). This patient showed an increase in serum total bilirubin up to 7.8 mg/dL and became asymptomatic in 4 weeks. The third patient did not show an HCV reactivation but developed a hepatic flare after chemotherapy was discontinued.

Pitini et al. recently described HCV reactivation and a hepatic flare in 10 patients with HCV infection and NHL treated with R-CHOP [38]. The hepatic flare was asymptomatic in 8 and symptomatic in 2 patients (Table 1): a 68-year-old male with DLBCL who developed HCV reactivation and a hepatic flare two weeks after the third cycle of R-CHOP and died of acute liver failure and a 65-year-old female with DLBCL who developed severe ascitis after four cycles of R-CHOP and died 1 week later for the uncontrolled progression of the underlying oncohaematological disease.

Similar results were reported by Tsutsumi et al. [37] in a prospective study of 4 patients with HCV infection and NHL treated with R-CHOP; all patients showed HCV reactivation and a hepatic flare during chemotherapy, but no severe clinical events occurred (Table 1).

In a retrospective study on 160 patients with Hodgkin's lymphoma and HCV infection, Arcaini et al. described hepatotoxicity in 5 (17.9%) of 28 patients with B-cell lymphoma treated with R-CHOP [43]. For 25 patients in this study, 15 treated with R-CHOP and 10 with CHOP, circulating HCV RNA was quantified at each cycle of chemotherapy; HCV-RNA quantification did not correlate to liver toxicity. 

Petrarca et al. treated HCV-associated mixed cryoglobulinaemia with rituximab and found this treatment useful and safe even in patients with a severe liver disease [44]; the patients in this study, however, had a low degree of immune depression and did not receive corticosteroids.

Worthy of mention is the literature showing a frequent occurrence of hepatic flares in patients receiving high-dose corticosteroids plus rituximab [36–40], probably because of a cumulative effect of these drugs, which both induce an increase in HCV replication, rituximab because of the impairment of antibody production, [18, 19, 45] and corticosteroids because of the enhancement of HCV entry to the hepatocytes as a consequence of an overexpression of specific receptors on the surface of these cells [46, 47]. 

### 1.2. Other Studies

Visco et al. reported an association between R-CHOP treatment for DLBCL and a mild increase in the liver enzyme level not requiring discontinuation of treatment in 5 (14%) of 35 patients [48]; this study, however, gave no information on HCV reactivation.

Coppola et al. described 28 anti-HCV/HCV RNA-negative patients receiving chemotherapy (rituximab based in 61% of cases) for oncohaematological diseases. These patients remained HCV RNA-negative both in plasma and PBMC and no hepatic flare was observed over an observation period of 6–24 months [49], most probably because no patient in this study had an occult HCV infection.

## 2. Conclusions

 Fragmentary information from case reports, small studies, and retrospective investigations suggest, on the whole, that rituximab-based chemotherapy favours viral replication in patients with HCV infection and oncohaematological diseases. Only a few small studies evaluated both variations in the HCV viral load and the development of a hepatic flare during or after R-CHOP treatment, but the results are concordant and strongly suggest a close association between these two events. A possible interpretation of this association is that R-CHOP induces an enhancement of HCV replication followed by a spontaneous subsequent decrease once treatment is reduced or discontinued. The consequent restoration of the immune control may induce a hepatic flare of varying clinical impact, that is, asymptomatic, symptomatic or life threatening, most probably reflecting the extent of cell-mediated hepatocellular necrosis. The clinical effects seem more evident in patients treated with a combination of rituximab and high-dose corticosteroids. 

The high prevalence worldwide of HCV infection in oncohaematological patients and the progressive increase in the use of R-CHOP for treating these diseases may create a heavy burden in the future for the health care authorities in several countries. 

## Figures and Tables

**Table 1:** Studies on rituximab-based chemotherapy that investigated both HCV reactivation and hepatic flares.

	Coppola et al., 2012 [40]	Ennishi et al., 2010 [36]	Marignani et al., 2011 [39]	Pitini et al., 2010 [38]	Tsutsumi et al., 2009 [37]
Type of study	Observational prospective	Multicentre retrospective	Observational retrospective	Observational prospective	Observational prospective
Disease	LNH, CLL	LNH (DLBCL)	LNH	LNH (DLBCL, FL)	LNH (DLBCL)
Number of cases	8	34	3	10	4
Treatment	R-CHOP in 5	R- and P-based	R-CHOP	RCHOP	R-CHOP in 1
	R-FC in 1	chemotherapy			R-CHO in 2
	CP and then R-FC				R-THP-CO in 1
	in 1				
	FC in 1				
With HCV reactivation, number of cases:	7	34	2	10	4
During CT	7	34	0	10	4
After CT	0	0	2	0	0
With hepatic flare, number of cases:	7	The available datum (27%) refers to flares observed in 131 patients, of whom only 34 were followed up for HCV RNA	2	10	3
During CT	7		0	10	3
After CT	0		2	0	0
				
				
				

NHL: non-Hodgkin lymphoma; DLBCL: diffuse large B-cell lymphoma; FL: follicular lymphoma; CLL: chronic lymphocytic leukaemia; R-CHO: rituximab, cyclophosphamide, doxorubicin, vincristine; RFC: rituximab, fludarabine, and cyclophosphamide; CP: cyclophosphamide, prednisone; FC: fludarabine and cyclophosphamide; R-THPCOP: rituximab, pirarubicin (tetrahydropyranyl-adriamicin [TPH] ) cyclophosphamid, vincristine without prednisone.
